# Supplementary material for: Overview of oral health status and associated risk factors in maritime settings: An updated systematic review
Source: PLoS One. 2023 Oct 18;18(10):e0293118. doi: 10.1371/journal.pone.0293118 (PMC10584167; doi:10.1371/journal.pone.0293118)
Supplement: S1 Fig — (DOCX) [file pone.0293118.s002.docx]

**S1 Figure: PRISMA 2020 flow diagram for new systematic reviews which included searches of databases, registers and other sources**

**Identification of studies via other methods**

**Identification of studies via databases and registers**

Records identified from:

Citation searching (n = 5)

etc.

Records removed *before screening*:

Duplicate records removed (n = 78)

Records marked as ineligible by automation tools (n = 85)

Records identified from*:

Databases (n = 255)

**Identification**

Records screened

(n = 92)

Records excluded**

(n = 54)

Reports sought for retrieval

(n = 5)

Reports sought for retrieval

(n = 38)

Reports not retrieved

(n = 4)

**Screening**

Reports assessed for eligibility

(n = 5)

Reports assessed for eligibility

(n = 34)

Reports excluded:

Reason 1: no relevant outcome (n = 6)

Reason 2: no relevant population (n = 9)

**Included**

Studies included in review

(n = 24)

*Consider, if feasible to do so, reporting the number of records identified from each database or register searched (rather than the total number across all databases/registers).

**If automation tools were used, indicate how many records were excluded by a human and how many were excluded by automation tools.

*From:*  Page MJ, McKenzie JE, Bossuyt PM, Boutron I, Hoffmann TC, Mulrow CD, et al. The PRISMA 2020 statement: an updated guideline for reporting systematic reviews. BMJ 2021;372:n71. doi: 10.1136/bmj.n71. For more information, visit: <http://www.prisma-statement.org/>
