# Supplementary material for: Overview of oral health status and associated risk factors in maritime settings: An updated systematic review
Source: PLoS One. 2023 Oct 18;18(10):e0293118. doi: 10.1371/journal.pone.0293118 (PMC10584167; doi:10.1371/journal.pone.0293118)
Supplement: S1 Table — (DOCX) [file pone.0293118.s003.docx]

**S1 Table: Characteristics of included studies on oral health status and associated factors in maritime settings**

| **Author,**  **Year of publication, country** | **Study**  **design** | **Population** | **Mean**  **Age/Age range** | **Sample** | **Period** | **Outcome** | **Result** |
| --- | --- | --- | --- | --- | --- | --- | --- |
| Varkey N.S [1]  India  2022 | CR | Fishermen | 28 | 200 | 3 months | Dental caries, oral hygiene status and aids, treatment needs | - Caries status: 82%  - OHI-S:  + Good: 23%  + Fair: 31%  + Poor: 46%  - Hygiene with toothbrush (43%), finger and toothpaste (32%), finger and tooth powder/salt (14%) & chewsticks (11%).  - Treatment need:  + Extraction (42.2%)  + Filling (33.9%)  + Pulp care (23.6%) |
| Tormeti D [2]  Ghana  2022 | CR | Fishermen | 20-70 | 138 | NA | Oral hygiene practice, plague index (PI), Community periodontal index of treatment need (CPITN) | - 34.9% cleaned their teeth twice daily.  - PI level 3: 100%  - CPITN: 100% with periodontal issues  + Level 4: 36.3%  + Level 5: 63.7% |
| Nithya V. R [3]  India  2021 | CR | Fishermen | 38 | 71 | NA | Oral hygiene, caries and gingiva status, oral mucosa lesions and associated factors | - Caries: 90.9%  - Gingiva disease: 97.2%  - Oral mucosa lesions prevalence is 20.8% with Leukoplakia (67.6%) and Ulcers (23.9%) are the most common types. Buccal mucosa is the most affected location (76%).  - Tobacco chewers: 62%  - Snuff: 54.9%  - Alcohol: 33.8%  - Smoker: 26.8%  Oral hygiene status:  - Average: 39.4%  - Poor: 60.6% |
| Schlagenhauf U [4]  Germany  2020 | RCT | Navy | 27 | 68  T: 33  Pla: 35 | 42 days | Effect of Probiotics on Bleeding on probing (BoP), gingival index (G.I), plaque control record (PCR), probing attachment level (PAL) and probing pocket depth (PPD) | BoP  - T reduced 27% at day 14  - T reduced 31% at day 42  - Pla reduced 4% at day 14  - Pla increased 5% at day 42  G.I  - T reduced 0.6 mean score at day 14  - T reduced 0.8 mean score at day 42  - Pla reduced 0.1 mean score at day 14  - Pla increased 0.1 mean score at day 42  PCR  - T reduced 8% at day 14  - T reduced 7% at day 42  - Pla increased 2% at day 14  - Pla increased 5% at day 42  PPD  - T reduced 0.2mm at day 14  - T reduced 0.3 mm at day 42  - Pla unchanged at day 14  - Pla increased 0.2mm at day 42    PAL  - T reduced 0.2mm at day 14  - T reduced 0.2 mm at day 42  - Pla increased 0.1 mm at day 14  - Pla increased 0.3mm at day 42 |
| Binaisse, P. [5]  France  2019 | RA | Seafarer  fishermen | 39 | 9122 | 4 years  2012-2016 | Dental emergency (DM) | % DM = 1.48%  DM reasons:  - Dental abscess: 51.8%  - Decayed: 33.3%  - Fracture: 8.8%  DM treatment: antibiotic, NSAID, antalgics and mouthwash. Antalgics is the most common medication in all cases. |
| Ugelvig Petersen, K. [6]  Denmark  2018 | CO  (RA) | Seafarer | NP | 44293 | 29 years | Oral cancer | Lip cancer: SIR (male) = 1.76 (S)  Oral cavity cancer: SIR (male) = 2.14 (S) |
| Singh, M. K. D. [7]  Malaysia  2018^(24)^ | CR | fishermen | 55.8 | 242 | 1 month | Oral health status and associated factors. | - Hygiene: brush 1/day (37.6%), 2/day (54.5%).  - Age, income, fishing type, additional occupation; frequency of pie and bun consumed, frequency of sweet and soft drink consumed are significantly associated with good oral health.  - Regular Smoker: 83.1%  - Regular coffee with sugar: 69.4% |
| Lodagala, A [8]  India  2018 | CR | Fishermen | 35-44 | 374 | 2 months | Dental caries status | - Tobacco user: 46.5%  - Non-tobacco user: 65.8% |
| Plotiansky, I. V. [9]  Ukraine  2018 | CR | Seafarer  fishermen | 22-60 | NP | NP | Quality of drinking water for seafarers | ﻿Fluctuation of chemical compositions.  Fluoride was absent in 75% ports. |
| Bhatt, Sumeet [10]  India  2017 | CR | Fishermen | 38.5 | 840 | 2 months | Oral health status & oral health risk factors | Caries status: 55%  Periodontal status: 99%  - Dental caries is associated with age, gender and education  - Periodontal health is associated with age, gender, education and income.  The perception of not having problem is the major barrier in seeking dental care.  The use of oral service  - Never visit dentists: 55.1%  - Reason to visit dentists:  Cleaning teeth: 17.2%  Tooth extraction: 69.2%  Filling: 10%  - Reason not to visit:  Do not have problem: 87.2%  - Treatment:  Extraction: 69.2% |
| Tarvainen, L, [11]  Nordic countries  2017 | RA | Seafarer  fishermen | 30-64 | Fishermen: 68309  Seafarer:  82127 | 45 years | Oral cancer | Tongue cancer  Seafarer (male): SIR= 1.66 (S);  Fishermen (male): SIR = 0.68 (NS)  Oral cavity cancer  Seafarer (male): SIR = 2 (S)  Fishermen (male): SIR = 0.94 (NS) |
| Marimoutou, Cyril [12]  France  2017 | RA | Seafarer | 37.7 | 10833 | 2012-2015 | Dental infection | Dental infection/total infection: 5/72 (8%)  Dental disease/total disease: 5/322 (1.5%) |
| Wang, G. [13]  China  2016 | RCT | Navy | 26.3 | 154  Ctr: 49  I1: 51  I2: 54 | 201 days | Effect of dental education and dental education & mouthwash on  - Debris status (DI-S)  - Calculus status (CIS)  - Gingiva bleeding status (SBI) | Control group  - DIS increased 22.5%(S)  - CIS increased 33.3%(S)  - SBI increased 82.86%(S)  Intervention 1 group  - DIS increased 22.5% (NS)  - CIS increased 33.3%(S)  - SBI increased 36.6%(S)  Intervention 2 group  - DIS unchanged (NS)  - CIS unchanged (NS)  - SBI decreased 58.33% (S) |
| Mahdi, S. S. [14]  Italy  2016 | CR | Seafarer | NP | 2060 | 4 months | Habits, attitude and problems regarding oral hygiene | Problems  - Smoke: 56.11%  - Alcohol: 11.45%  - Dairy product: 55.67%  - Full-set of teeth: 61%  - 82% lose teeth due to extraction  Hygiene habit  - Brush daily: 74%  - Brush 2/day: 40.48% |
| Anzil, K. [15]  India  2016 | CR | Fishermen | 15-54 | 362 | NP | Oral habits and oral mucosal lesions prevalence | Abnormal mucosa: 14.9%  Ulceration: 4.9%  Leukoplakia: 1.3%  Abscess: 1.3%  Oral habits  Alcohol: 48.8%  Smoke: 24.3%  Chewed tobacco: 32% |
| Aapaliya, P. [16]  India  2015^(28)^ | CR | Seafarer | 21-70 | 385 | 2 months | Dental caries and periodontal condition | Dental caries: 88%  Periodontal disease: 75.1%  Dental calculus: 33.7%  Periodontal pocket 4-5mm: 20%  Gingiva bleeding: 11.6%  Periodontal pocket > 6mm: 9.8%  Oral hygiene  Seaweed: 59.2%  Toothbrush and toothpaste: 18.9%  Finger and toothpaste:15.5%  Unhealthy habits  Smoking: 25%  Smokeless tobacco: 14.5%  Alcohol consumption: 14.2% |
| Kumar, D. R. [17]  India  2015 | CR | Fishermen | >60 | 1200 | 2 months | Prosthetic status and need | Prosthetic status:  No prosthesis: 76.7%  Full-denture: 12.3%  Prosthetic treatment need: 65.2%  Multi-unit prosthetic: 27.3%  Full-prosthetic: 23.6% |
| ﻿Gunepin, Mathieu [18]  France  2015 | CR | Military diver | NP | 1317 | NP | Dental barotrauma | Prevalence: 5.3%  34.3% DB disrupted diving  Consequences:  Fracture & loss dental restoration (68.6%)  Fracture: 15.7%  Loosening fixed prosthesis: 11.4% |
| de Oliveira Ribeiro, Artur et al [19]  2014  Brazil | CR | Fishermen | 42 | 210 | 8 months | Actinic cheilitis (AC) prevalence and associated factors | % AC = 11.4%  -  The AC prevalence is higher among those with  - Fair skin type  - Age > 50  - Cumulative solar radiation exposure > 30 years  - Daily solar radiation exposure > 4 hours |
| Asawa, K. [20]  India  2014 | CR | Fishermen | 33.5 | 1100 | 6 months | Oral health status | Caries: 82.6%  Periodontal diseases: 85.4%  Treatment need  Extraction: 66.2%  Pulp treatment: 63.9%  Oral Hygiene instrument  Chew stick: 43.1%  Brushing: 24% |
| Von Wilmowsky, C. [21]  Germany  2014 | RA | Navy | 25 | 650 | 2 months | Oral health status and evaluate dental service | Treatment need: 10.9%  Emergency: 3.69% (147.7 cases/1000 soldiers/year)  Treatment types:  Pulp restoration: 50%  Dental filling: 41.6%  Tooth extraction: 4.1% |
| Chandroth, S. V. [22]  India  2014^(33)^ | CR | Fishermen | 18-68 | 979 | 2 months | Oral mucosa lesions prevalence | Prevalence: 30%  Leukoplakia: 13.8%  Ulceration: 7.2%  - Oral hygiene  Chew stick: 42.9%  Finger: 29.9%  Brushing: 23.9%  - Oral habits  Smoke: 20%  smokeless tobacco: 21.9%  Alcohol: 15.4% |
| Elmer, T. B. [23]  UK  2011 | CR | Navy | 20 | 170 | NP | Caries status and treatment need | Mean decayed teeth: 1.93  Treatment need: 56.8% |
| Piñera-Marques, K. [24]  Brazil  2010 | CO | Fishermen | 50.6 | 125 | NP | Lower lip lesions (AC) | Lower lip lesion: 12.8%  Malignant: 3.2%  Alcohol: 32%  Smoking: 38.4% |
| S: significant; NS: non-significant; NP: not applicable; CR: cross-sectional study; CO: cohort study; RCT: randomized controlled study; RA: retrospective analysis; DM: dental emergency; NSAID: non-steroid anti-inflammatory drug; SIR: standardized incidence ratio; ctr: control group; I1: intervention 1 with only dental education; I2: intervention 2 with dental education and mouthwash; DI-S: debris index status; CI-S: calculus index status; SBI: sulcus bleeding index; AC: actinic cheilitis. BoP: Bleeding on probing, G.I: gingival index, PCR: plaque control record, PAL: probing attachment level, PPD: probing pocket depth, SIR: standardized incidence rate. | | | | | | | |

**Reference**

1. N.S V, Vas R, Uppala H, Vas N, Jalihal S, Ankola A, et al. Dental caries, oral hygiene status and treatment needs of fishermen and non-fishermen population in South Goa, India. International Maritime Health [Internet]. 2022 2022-10-10 [cited 2022 Dec 10]; 73(3):[125-32 pp.]. Available from: <https://doi.org/10.5603/IMH.2022.0025>.

2. Tormeti D, Nii-Aponsah H, Sackeyfio J, Blankson PK, Quartey-Papafio N, Arthur M, et al. Periodontal status and oral hygiene practices among adults in a peri-urban fishing community in Ghana. Pan African Medical Journal [Internet]. 2022 [cited 2022 Dec 20]; 42:[126 p.]. Available from: <https://dx.doi.org/10.11604/pamj.2022.42.126.24557>.

3. Nithya VR KC, Sridhar C, Arumugam AE. Assessment of Oral Health Care Needs among Fishermen Living in North Chennai, India – A Cross Sectional Study2021 15 December 2021 [cited 2022 Dec 2]. Available from: <http://eprints.asianrepository.com/id/eprint/1186/1/34214-Article%20Text-61558-1-10-20211229.pdf>.

4. Schlagenhauf U, Rehder J, Gelbrich G, Jockel-Schneider Y. Consumption of Lactobacillus reuteri-containing lozenges improves periodontal health in navy sailors at sea: A randomized controlled trial. Journal of Periodontology [Internet]. 2020 2020/10/01 [cited 2022 Dec 10]; 91(10):[1328-38 pp.]. Available from: <https://doi.org/10.1002/JPER.19-0393>.

5. Binaisse P, Dehours E, Bodere C, Chevalier V, Le Fur Bonnabesse A. Dental emergencies at sea: A study in the French maritime TeleMedical Assistance Service. J Telemed Telecare [Internet]. 2019 Jan 15 [cited 2020 July 2]:[1357633x18818736 p.]. Available from: <https://doi.org/10.1177/1357633X18818736>.

6. Ugelvig Petersen K, Volk J, Kaerlev L, Lyngbeck Hansen H, Hansen J. Cancer incidence among merchant seafarers: an extended follow-up of a Danish cohort. Occup Environ Med [Internet]. 2018 Aug [cited 2022 Aug 2]; 75(8):[582-5 pp.]. Available from: <https://oem.bmj.com/content/75/8/582>.

7. Singh MKD, Abdulrahman SA, Rashid A. Assessment of oral health status and associated lifestyle factors among Malaysian Fishermen in Teluk Bahang, Penang: An analytical cross-sectional study. Indian J Dent Res [Internet]. 2018 May-Jun [cited 2022 May 4]; 29(3):[378-90 pp.]. Available from: <https://doi.org/10.4103/ijdr.IJDR_545_17>.

8. Lodagala A, Pachava S, Talluri D, Chandu V. Association between tobacco usage and dental caries among 35-44-year-old fishermen of North Coastal Region of South Indian State, Andhra Pradesh. Journal of Indian Association of Public Health Dentistry [Internet]. 2018 October 1, 2018 [cited 2022 Dec 1]; 16(4):[308-12 pp.]. Available from: <http://www.jiaphd.org/article.asp?issn=2319-5932;year=2018;volume=16;issue=4;spage=308;epage=312;aulast=Lodagala>.

9. Plotiansky IV BN, Stets NV, . Impact of drinking water quality on the occurrence of and the development of dental diseases in seafarers of overseas navigation. [Internet]. 2018 [cited 2022 Dec 28]; 8(1). Available from: <https://apcz.umk.pl/JEHS/article/view/5619>.

10. Bhatt S, Rajesh G, Rao A, Shenoy R, Pai M, Nayak V. Factors influencing Oral Health and Utilization of Oral Health Care in an Indian Fishing Community, Mangaluru City, India. World Journal of Dentistry [Internet]. 2017 07/01 [cited 2020 Jan 5]; 8:[321-6 pp.]. Available from: <http://dx.doi.org/10.5005/jp-journals-10015-1458>.

11. Tarvainen L, Suojanen J, Kyyronen P, Lindqvist C, Martinsen JI, Kjaerheim K, et al. Occupational Risk for Oral Cancer in Nordic Countries. Anticancer Res [Internet]. 2017 Jun [cited 2022 Dec 12]; 37(6):[3221-8 pp.]. Available from: <https://doi.org/10.21873/anticanres.11684>.

12. Marimoutou C, Tufo D, Chaudet H, Abdul Samad M, Gentile G, Drancourt M. Infection burden among medical events onboard cargo ships: a four-year study. Journal of Travel Medicine [Internet]. 2017 [cited 2022 Dec 4]; 24(3). Available from: <https://doi.org/10.1093/jtm/tax010>.

13. Wang G, Li W, Liu Y, Chen X, Huang J, Zhao Y, et al. Efficacy of dental health education and a novel mouthwash on periodontal health of navy personnel on a long ocean-going training mission. International Journal of Clinical and Experimental Medicine [Internet]. 2016 [cited 2022; 9(8):[16653-60 pp.]. Available from: <https://www.scopus.com/inward/record.uri?eid=2-s2.0-84985952049&partnerID=40&md5=d3c8cb61065e1b1b6433bf1499107f37>.

14. Mahdi SS, Sibilio F, Amenta F. Dental hygiene habits and oral health status of seafarers. International maritime health [Internet]. 2016 [cited 2020 February 2]; 67(1):[9-13 pp.]. Available from: <https://www.scopus.com/inward/record.uri?eid=2-s2.0-85007128505&doi=10.5603%2fIMH.2016.0003&partnerID=40&md5=b3e88238f9bf93794c51489030a5e25b>.

15. Anzil K, Mathews J, Sai AG, Kiran M, Kevin S, Sunith S. Prevalence of Deleterious Oral Habits and Oral Mucosal Lesions among Fishermen Population of Mahe, South India. J Contemp Dent Pract [Internet]. 2016 Sep 1 [cited 2020 Feb 2]; 17(9):[745-9 pp.]. Available from: <https://doi.org/10.5005/jp-journals-10024-1923>.

16. Aapaliya P, Shinde K, Deswal AK, Mohapatra S, Saleem S, Mangal R, et al. Assessment of oral health among seafarers in Mundra Port, Kutch, Gujarat: a cross-sectional study. International maritime health [Internet]. 2015 [cited 2022 Jan 3]; 66(1):[11-7 pp.]. Available from: <https://www.scopus.com/inward/record.uri?eid=2-s2.0-84952313729&doi=10.5603%2fIMH.2015.0004&partnerID=40&md5=0553e7b25b0fc2585f47540c66ec8db5>.

17. Kumar DR, Raju DS, Naidu L, Deshpande S, Chadha M, Agarwal A. Prosthetic status and prosthetic needs amongst geriatric fishermen population of Kutch coast, Gujarat, India. Roczniki Państwowego Zakładu Higieny [Internet]. 2015 [cited 2022 Dec 5]; 66(2):[167-71 pp.]. Available from: <https://www.scopus.com/inward/record.uri?eid=2-s2.0-84953343744&partnerID=40&md5=6fa32665a752998e51edc1ce9a5f2625>.

18. Gunepin M, Derache F, Dychter D.D.S L, Blatteau J-E, Nakdimon I, Zadik Y. Dental Barotrauma in French Military Divers: Results of the POP Study. Aviation Space and Environmental Medicine [Internet]. 2015 07/01 [cited 2022 Dec 2]; 86:[652-5 pp.]. Available from: <https://doi.org/10.3357/AMHP.4197.2015>.

19. de Oliveira Ribeiro A, da Silva LCF, Martins-Filho PRS. Prevalence of and risk factors for actinic cheilitis in Brazilian fishermen and women. International Journal of Dermatology [Internet]. 2014 2014/11/01 [cited 2022 Dec 20]; 53(11):[1370-6 pp.]. Available from: <https://doi.org/10.1111/ijd.12526>.

20. Asawa K, Pujara P, Tak M, Nagarajappa R, Aapaliya P, Bhanushali N, et al. Oral health status of fishermen and non-fishermen community of Kutch district, Gujarat, India: a comparative study. International maritime health [Internet]. 2014 [cited 2022 Dec 4]; 65(1):[1-6 pp.]. Available from: <https://www.scopus.com/inward/record.uri?eid=2-s2.0-84921317341&doi=10.5603%2fMH.2014.0001&partnerID=40&md5=c5d5ea5de47294bc769ec63bee05fe14>.

21. von Wilmowsky C, Kiesewetter MR, Moest T. Dental treatment on a German warship during a three-month deployment. J R Army Med Corps [Internet]. 2014 Mar [cited 2022 Mar 5]; 160(1):[42-5 pp.]. Available from: <https://doi.org/10.1136/jramc-2013-000063>.

22. Chandroth SV, Venugopal HK, Puthenveetil S, Jayaram A, Mathews J, Suresh N, et al. Prevalence of oral mucosal lesions among fishermen of Kutch coast, Gujarat, India. International maritime health [Internet]. 2014 [cited 2022 Nov 12]; 65(4):[192-8 pp.]. Available from: <https://www.scopus.com/inward/record.uri?eid=2-s2.0-84939440060&doi=10.5603%2fIMH.2014.0037&partnerID=40&md5=9f72609572acab528d497671d0e1dab4>.

23. Elmer TB, Langford J, McCormick R, Morris AJ. Is there a differential in the dental health of new recruits to the British Armed Forces? A pilot study. Br Dent J [Internet]. 2011 Nov 11 [cited 2022 Nov 11]; 211(9):[E18 p.]. Available from: <https://doi.org/10.1038/sj.bdj.2011.937>.

24. Piñera-Marques K, Lorenço SV, Silva LF, Sotto MN, Carneiro PC. Actinic lesions in fishermen's lower lip: clinical, cytopathological and histopathologic analysis. Clinics (Sao Paulo) [Internet]. 2010 Apr [cited 2022 Dec 4]; 65(4):[363-7 pp.]. Available from: <https://doi.org/10.1590/S1807-59322010000400003>.
