# Supplementary material for: Overview of oral health status and associated risk factors in maritime settings: An updated systematic review
Source: PLoS One. 2023 Oct 18;18(10):e0293118. doi: 10.1371/journal.pone.0293118 (PMC10584167; doi:10.1371/journal.pone.0293118)
Supplement: S1 Appendix — (DOCX) [file pone.0293118.s004.docx]

**S1 Appendix: Search Strategy**

| **PubMed** |
| --- |
| ((((((((seafarer[Title/Abstract]) OR (fishermen[Title/Abstract])) OR (sailer[Title/Abstract])) OR (shipper[Title/Abstract])) OR (mariner[Title/Abstract])) OR (sailor[Title/Abstract])) OR (seagoing[Title/Abstract])) OR (navigator[Title/Abstract])) AND (((((((((((((("oral health"[MeSH Terms]) OR ("oral health"[Title/Abstract])) OR ("mouth diseases"[MeSH Terms])) OR ("mouth diseases"[Title/Abstract])) OR ("oral hygiene"[MeSH Terms])) OR ("oral hygiene"[Title/Abstract])) OR ("mouth neoplasm "[Title/Abstract])) OR ("mouth neoplasm"[MeSH Terms])) OR ("dental caries"[MeSH Terms])) OR ("dental caries"[Title/Abstract])) OR ("periodontal diseases"[Title/Abstract])) OR ("periodontal diseases"[MeSH Terms])) OR ("dental plaque"[MeSH Terms])) OR ("dental plaque"[Title/Abstract])) |
| **Scopus** |
| ( ( TITLE-ABS-KEY ( seafarer )  OR  TITLE-ABS-KEY ( fishermen )  OR  TITLE-ABS-KEY ( seamen )  OR  TITLE-ABS-KEY ( mariner )  OR  TITLE-ABS-KEY ( sailer )  OR  TITLE-ABS-KEY ( shipper )  OR  TITLE-ABS-KEY ( navigator ) ) )  AND  ( ( TITLE-ABS-KEY ( "oral health" )  OR  TITLE-ABS-KEY ( "mouth diseases" )  OR  TITLE-ABS-KEY ( "oral hygiene" )  OR  TITLE-ABS-KEY ( "dental caries" )  OR  TITLE-ABS-KEY ( "periodontal diseases" )  OR  TITLE-ABS-KEY ( "oral cancer" )  OR  TITLE-ABS-KEY ( "dental plaque" ) ) ) |
| **Ovid (Embase)** |
| sailor/seafarer.mp. OR fishermen.mp. or fisherman/ or fishing/ OR mariner.mp. or sailor/ OR shipper.mp. OR navigator.mp. And "oral health".mp. OR mouth disease/ep [Epidemiology] OR mouth cancer/ep [Epidemiology] OR dental caries/ep [Epidemiology] OR periodontal disease/ep [Epidemiology] OR "oral hygiene".mp. OR tooth plaque/ep [Epidemiology] |
| **CINAHL** |
| AB ( AB seafarer OR AB fishermen OR AB mariner OR AB shipper OR AB seamen OR AB sailors OR AB navigator ) AND AB ( AB "oral health" OR AB "mouth diseases" OR AB "oral cancer" OR AB "dental caries" OR AB "periodontal diseases" OR AB "oral hygiene" OR AB "dental plaque" ) |
| **Web of Science** |
| Search 1:  seafarer (Topic) or seamen (Topic) or fishermen (Topic) or mariner (Topic) or sailor (Topic) or shipper (Topic)  Search 2: TOPIC: ("oral health") OR TOPIC: ("mouth diseases") OR TOPIC: ("oral cancer") OR TOPIC: ("dental caries") OR TOPIC: ("periodontal diseases") OR TOPIC: ("dental plaque") OR TOPIC: ("oral hygiene")  Search 3: #1 AND #2 |
