# Supplementary material for: Overview of oral health status and associated risk factors in maritime settings: An updated systematic review
Source: PLoS One. 2023 Oct 18;18(10):e0293118. doi: 10.1371/journal.pone.0293118 (PMC10584167; doi:10.1371/journal.pone.0293118)
Supplement: S2 Appendix — (DOCX) [file pone.0293118.s005.docx]

**S2 Appendix Quality assessment of the observational studies**

| **Author** | **Item _1_** | **Item _2_** | **Item 3** | **Item 4** | **Item 5** | **Item 6** | **Item 7** | **Item 8** | **Item 9** | **Total** |
| --- | --- | --- | --- | --- | --- | --- | --- | --- | --- | --- |
| Varkey N.S et al, 2022 [1] | Y | Y | Y | Y | N | Y | Y | Y | Y | 8/9 |
| Tormeti D et al, 2022 [2] | Y | Y | Un | Y | Y | Y | Un | Y | Y | 7/9 |
| V. R. Nithya et al, 2021 [3] | Y | Un | Un | Y | Un | Y | Un | Y | Y | 5/9 |
| ﻿Pierre Binaisse, 2019 [4] | Y | Y | Y | Y | Y | Y | Y | N | Y | 8/9 |
| ﻿Kajsa Ugelvig Petersen, 2018 [5] | Y | Y | Y | Y | Y | Y | Y | Y | N | 8/9 |
| ﻿Manjit Kaur Doola Singh, 2018 [6] | Y | Y | Y | Y | Y | Y | Y | Y | Y | 9/9 |
| ﻿Asha Lodagala, 2018 [7] | Y | Y | Un | Un | Y | Y | Y | Y | Y | 7/9 |
| ﻿Sumeet Bhatt, 2017 [8] | Y | Y | Y | Y | Y | Y | Y | Y | Y | 9/9 |
| ﻿Laura Tarvainen, 2017 [9] | Y | Y | Y | Y | Un | Y | Un | Y | Un | 6/9 |
| ﻿Syed Sarosh Mahdi 2016 [10] | N | Y | Y | Un | Un | Y | Y | Un | Un | 4/9 |
| ﻿KSA Anzi, 2016 [11] | Y | Y | Y | Y | Y | Y | Y | Un | Y | 8/9 |
| ﻿Pankaj Aapaliya, 2015 [12] | Y | Un | Un | Y | Y | Y | Y | Y | Y | 7/9 |
| ﻿Dola Rama Venkata Kumar, 2015 [13] | Y | Y | Y | Y | Y | Y | Y | Y | Y | 9/9 |
| de Oliveira Ribeiro A et al, 2014 [14] | Y | Y | Y | Y | Y | Y | Y | Y | Y | 9/9 |
| ﻿Kailash Asawa, 2014 [15] | Y | Y | Y | Y | Y | Y | Y | Y | Y | 9/9 |
| ﻿Cornelius von Wilmowsky, 2014 [16] | Y | Un | Y | Un | Un | Y | Y | Un | Y | 5/9 |
| ﻿Santhosh Vediyera Chandroth, 2014 [17] | Y | Y | Y | Y | Y | Y | Y | Y | Y | 9/9 |
| ﻿T. B. Elmer, 2011 [18] | Un | Un | Y | Y | N | Y | Y | Un | Y | 5/9 |
| ﻿Piñera-Marques, 2010 [19] | Y | NA | Un | Y | Y | Y | Y | Y | Y | 7/9 |
| ﻿I. V. Plotiansky, 2018 [20] | Y | Un | Un | Un | Un | Y | Un | Un | Un | 2/9 |
| ﻿Cyril Marimoutou, 2017 [21] | Y | Y | N | Y | Y | Y | Y | Y | Y | 8/9 |
| ﻿Mathieu Gunepin, 2015 [22] | Y | Y | Y | Y | Un | Un | Un | Un | Y | 5/9 |
| **Y:** Yes**, N:** No**, Un:** Unclear**.**  **Item_1_:** Was the sample frame appropriate to address the target population? **Item_2_:** Were study participants sampled in an appropriate way?  **Item_3_**: Was the sample size adequate?**;** **Item_4_**: Were the study subjects and the setting described in detail?**;** **Item_5_**: Was the data analysis conducted with sufficient coverage of the identified sample?**;** **Item_6_**: Were valid methods used for the identification of the condition?**;** **Item_7_**: Was the condition measured in a standard, reliable way for all participants?**;** **Item_8_**: Was there appropriate statistical analysis?**;** **Item_9_**: Was the response rate adequate, and if not, was the low response rate managed appropriately? | | | | | | | | | | |

**Reference**

1. N.S V, Vas R, Uppala H, Vas N, Jalihal S, Ankola A, et al. Dental caries, oral hygiene status and treatment needs of fishermen and non-fishermen population in South Goa, India. International Maritime Health [Internet]. 2022 2022-10-10 [cited 2022 Dec 10]; 73(3):[125-32 pp.]. Available from: <https://doi.org/10.5603/IMH.2022.0025>.

2. Tormeti D, Nii-Aponsah H, Sackeyfio J, Blankson PK, Quartey-Papafio N, Arthur M, et al. Periodontal status and oral hygiene practices among adults in a peri-urban fishing community in Ghana. Pan African Medical Journal [Internet]. 2022 [cited 2022 Dec 20]; 42:[126 p.]. Available from: <https://dx.doi.org/10.11604/pamj.2022.42.126.24557>.

3. Nithya VR KC, Sridhar C, Arumugam AE. Assessment of Oral Health Care Needs among Fishermen Living in North Chennai, India – A Cross Sectional Study2021 15 December 2021 [cited 2022 Dec 2]. Available from: <http://eprints.asianrepository.com/id/eprint/1186/1/34214-Article%20Text-61558-1-10-20211229.pdf>.

4. Binaisse P, Dehours E, Bodere C, Chevalier V, Le Fur Bonnabesse A. Dental emergencies at sea: A study in the French maritime TeleMedical Assistance Service. J Telemed Telecare [Internet]. 2019 Jan 15 [cited 2020 July 2]:[1357633x18818736 p.]. Available from: <https://doi.org/10.1177/1357633X18818736>.

5. Ugelvig Petersen K, Volk J, Kaerlev L, Lyngbeck Hansen H, Hansen J. Cancer incidence among merchant seafarers: an extended follow-up of a Danish cohort. Occup Environ Med [Internet]. 2018 Aug [cited 2022 Aug 2]; 75(8):[582-5 pp.]. Available from: <https://oem.bmj.com/content/75/8/582>.

6. Singh MKD, Abdulrahman SA, Rashid A. Assessment of oral health status and associated lifestyle factors among Malaysian Fishermen in Teluk Bahang, Penang: An analytical cross-sectional study. Indian J Dent Res [Internet]. 2018 May-Jun [cited 2022 May 4]; 29(3):[378-90 pp.]. Available from: <https://doi.org/10.4103/ijdr.IJDR_545_17>.

7. Lodagala A, Pachava S, Talluri D, Chandu V. Association between tobacco usage and dental caries among 35-44-year-old fishermen of North Coastal Region of South Indian State, Andhra Pradesh. Journal of Indian Association of Public Health Dentistry [Internet]. 2018 October 1, 2018 [cited 2022 Dec 1]; 16(4):[308-12 pp.]. Available from: <http://www.jiaphd.org/article.asp?issn=2319-5932;year=2018;volume=16;issue=4;spage=308;epage=312;aulast=Lodagala>.

8. Bhatt S, Rajesh G, Rao A, Shenoy R, Pai M, Nayak V. Factors influencing Oral Health and Utilization of Oral Health Care in an Indian Fishing Community, Mangaluru City, India. World Journal of Dentistry [Internet]. 2017 07/01 [cited 2020 Jan 5]; 8:[321-6 pp.]. Available from: <http://dx.doi.org/10.5005/jp-journals-10015-1458>.

9. Tarvainen L, Suojanen J, Kyyronen P, Lindqvist C, Martinsen JI, Kjaerheim K, et al. Occupational Risk for Oral Cancer in Nordic Countries. Anticancer Res [Internet]. 2017 Jun [cited 2022 Dec 12]; 37(6):[3221-8 pp.]. Available from: <https://doi.org/10.21873/anticanres.11684>.

10. Mahdi SS, Sibilio F, Amenta F. Dental hygiene habits and oral health status of seafarers. International maritime health [Internet]. 2016 [cited 2020 February 2]; 67(1):[9-13 pp.]. Available from: <https://www.scopus.com/inward/record.uri?eid=2-s2.0-85007128505&doi=10.5603%2fIMH.2016.0003&partnerID=40&md5=b3e88238f9bf93794c51489030a5e25b>.

11. Anzil K, Mathews J, Sai AG, Kiran M, Kevin S, Sunith S. Prevalence of Deleterious Oral Habits and Oral Mucosal Lesions among Fishermen Population of Mahe, South India. J Contemp Dent Pract [Internet]. 2016 Sep 1 [cited 2020 Feb 2]; 17(9):[745-9 pp.]. Available from: <https://doi.org/10.5005/jp-journals-10024-1923>.

12. Aapaliya P, Shinde K, Deswal AK, Mohapatra S, Saleem S, Mangal R, et al. Assessment of oral health among seafarers in Mundra Port, Kutch, Gujarat: a cross-sectional study. International maritime health [Internet]. 2015 [cited 2022 Jan 3]; 66(1):[11-7 pp.]. Available from: <https://www.scopus.com/inward/record.uri?eid=2-s2.0-84952313729&doi=10.5603%2fIMH.2015.0004&partnerID=40&md5=0553e7b25b0fc2585f47540c66ec8db5>.

13. Kumar DR, Raju DS, Naidu L, Deshpande S, Chadha M, Agarwal A. Prosthetic status and prosthetic needs amongst geriatric fishermen population of Kutch coast, Gujarat, India. Roczniki Państwowego Zakładu Higieny [Internet]. 2015 [cited 2022 Dec 5]; 66(2):[167-71 pp.]. Available from: <https://www.scopus.com/inward/record.uri?eid=2-s2.0-84953343744&partnerID=40&md5=6fa32665a752998e51edc1ce9a5f2625>.

14. de Oliveira Ribeiro A, da Silva LCF, Martins-Filho PRS. Prevalence of and risk factors for actinic cheilitis in Brazilian fishermen and women. International Journal of Dermatology [Internet]. 2014 2014/11/01 [cited 2022 Dec 20]; 53(11):[1370-6 pp.]. Available from: <https://doi.org/10.1111/ijd.12526>.

15. Asawa K, Pujara P, Tak M, Nagarajappa R, Aapaliya P, Bhanushali N, et al. Oral health status of fishermen and non-fishermen community of Kutch district, Gujarat, India: a comparative study. International maritime health [Internet]. 2014 [cited 2022 Dec 4]; 65(1):[1-6 pp.]. Available from: <https://www.scopus.com/inward/record.uri?eid=2-s2.0-84921317341&doi=10.5603%2fMH.2014.0001&partnerID=40&md5=c5d5ea5de47294bc769ec63bee05fe14>.

16. von Wilmowsky C, Kiesewetter MR, Moest T. Dental treatment on a German warship during a three-month deployment. J R Army Med Corps [Internet]. 2014 Mar [cited 2022 Mar 5]; 160(1):[42-5 pp.]. Available from: <https://doi.org/10.1136/jramc-2013-000063>.

17. Chandroth SV, Venugopal HK, Puthenveetil S, Jayaram A, Mathews J, Suresh N, et al. Prevalence of oral mucosal lesions among fishermen of Kutch coast, Gujarat, India. International maritime health [Internet]. 2014 [cited 2022 Nov 12]; 65(4):[192-8 pp.]. Available from: <https://www.scopus.com/inward/record.uri?eid=2-s2.0-84939440060&doi=10.5603%2fIMH.2014.0037&partnerID=40&md5=9f72609572acab528d497671d0e1dab4>.

18. Elmer TB, Langford J, McCormick R, Morris AJ. Is there a differential in the dental health of new recruits to the British Armed Forces? A pilot study. Br Dent J [Internet]. 2011 Nov 11 [cited 2022 Nov 11]; 211(9):[E18 p.]. Available from: <https://doi.org/10.1038/sj.bdj.2011.937>.

19. Piñera-Marques K, Lorenço SV, Silva LF, Sotto MN, Carneiro PC. Actinic lesions in fishermen's lower lip: clinical, cytopathological and histopathologic analysis. Clinics (Sao Paulo) [Internet]. 2010 Apr [cited 2022 Dec 4]; 65(4):[363-7 pp.]. Available from: <https://doi.org/10.1590/S1807-59322010000400003>.

20. Plotiansky IV BN, Stets NV, . Impact of drinking water quality on the occurrence of and the development of dental diseases in seafarers of overseas navigation. [Internet]. 2018 [cited 2022 Dec 28]; 8(1). Available from: <https://apcz.umk.pl/JEHS/article/view/5619>.

21. Marimoutou C, Tufo D, Chaudet H, Abdul Samad M, Gentile G, Drancourt M. Infection burden among medical events onboard cargo ships: a four-year study. Journal of Travel Medicine [Internet]. 2017 [cited 2022 Dec 4]; 24(3). Available from: <https://doi.org/10.1093/jtm/tax010>.

22. Gunepin M, Derache F, Dychter D.D.S L, Blatteau J-E, Nakdimon I, Zadik Y. Dental Barotrauma in French Military Divers: Results of the POP Study. Aviation Space and Environmental Medicine [Internet]. 2015 07/01 [cited 2022 Dec 2]; 86:[652-5 pp.]. Available from: <https://doi.org/10.3357/AMHP.4197.2015>.
