# Supplementary material for: Overview of oral health status and associated risk factors in maritime settings: An updated systematic review
Source: PLoS One. 2023 Oct 18;18(10):e0293118. doi: 10.1371/journal.pone.0293118 (PMC10584167; doi:10.1371/journal.pone.0293118)
Supplement: S3 Appendix — (DOCX) [file pone.0293118.s006.docx]

**S3 Appendix: Quality assessment of RCT**

| **Author** | **Domain 1** | **Domain 2** | | **Domain 3** | **Domain 4** | **Domain 5** | **Overall risk of bias** |
| --- | --- | --- | --- | --- | --- | --- | --- |
|  |  | **Effect of assignments to intervention** | **Effect of adhering to intervention** |  |  |  |  |
| Schlagenhauf U, 2020 [1] | L | L | SC | L | L | L | SC |
| Guodong Wang, 2016 [2] | SC | L | L | L | L | L | SC |
| L: low risk of bias; SC: some concern risk of bias; Domain 1: Risk of bias arising from the randomization process; Domain 2: Risk of bias due to deviations from the intended interventions; Domain 3: Missing outcome data; Domain 4: Risk of bias in Measurement of the outcome; Domain 5: risk of bias in selection of the reported result. | | | | | | | |

**Reference**

1. Schlagenhauf U, Rehder J, Gelbrich G, Jockel-Schneider Y. Consumption of Lactobacillus reuteri-containing lozenges improves periodontal health in navy sailors at sea: A randomized controlled trial. Journal of Periodontology [Internet]. 2020 2020/10/01 [cited 2022 Dec 10]; 91(10):[1328-38 pp.]. Available from: <https://doi.org/10.1002/JPER.19-0393>.

2. Wang G, Li W, Liu Y, Chen X, Huang J, Zhao Y, et al. Efficacy of dental health education and a novel mouthwash on periodontal health of navy personnel on a long ocean-going training mission. International Journal of Clinical and Experimental Medicine [Internet]. 2016 [cited 2022; 9(8):[16653-60 pp.]. Available from: <https://www.scopus.com/inward/record.uri?eid=2-s2.0-84985952049&partnerID=40&md5=d3c8cb61065e1b1b6433bf1499107f37>.
