# Supplementary material for: Overview of oral health status and associated risk factors in maritime settings: An updated systematic review
Source: PLoS One. 2023 Oct 18;18(10):e0293118. doi: 10.1371/journal.pone.0293118 (PMC10584167; doi:10.1371/journal.pone.0293118)
Supplement: Attachment — Submitted filename: Response to Reviewer.docx [file pone.0293118.s007.docx]

**Rebuttal letter**

We thank you reviewer for the thoughtful inputs to our manuscript. In the revised version, we tried to address all raised points. Below, you will find point-by-point reply, including line numbers in new revised manuscripts that correspond with each raised point.

**Reviewer 1**

**Question**: As the date of the latest search among selected databases is Jan/22, please update the search until August/22.

**Answer**: thank you for this suggestion, we updated the search date to August 2022.

**Question**: Line 155-161- For the potentially malignant disorders, can you further discuss them? (eg. leukoplakia and actinic cheilitis). Is there any follow-up of these lesions?

**Answer**: for the potentially malignant disorders, we have already inputted some more details with data of Leukoplakia, ulceration and actinic cheilitis. No data about follow-up was found from included studies. (line 182)

**Question**: Line 156 - Please describe the findings related to oral cancer, such as the risk of developing (percentage) compared to healthy people.
**Answer**: we have included more detail about the risk of developing oral cancer of seafarer (standardized incidence ratio) compared to healthy population – (line 180)

**Question**: Line 160-161 - Please provide the prevalence of ulcerations and leukoplakias in percentage.

**Answer**: we have provided percentage of Leukoplakia and ulceration into the manuscript (Line 187)

**Question**: Line 170 - Five from 322 (percentage?)

**Answer**: we have switched to percentage (Line 199)

**Question**: Line 229 - ... dental outpatients in India (percentage?)

**Answer**: we have added the percentage of dental outpatients in India (Line 274)

**Question**: Line 238 - 239 - How much higher?

**Answer**: we have added the percentage of other population (Line 286)

**Question**: Line 253-254 - ... health education does (not?) affect protection of periodontal.

**Answer**: we have revised the sentence to make the meaning more explicit. (Line 309)

**Question**: Figure 1 - Please use the latest version of the PRISM flow diagram (2020)

**Answer**: we have changed to the new version of Prima glow diagram 2020. (Page 23)

**Question**: Appendix S1 - Can you provide the search strategy for the Web of Science database?

**Answer**: we have added the search strategy of Web of Science database on the S1 Appendix.

**Reviewer 2**

Feedback:

“The authors present a well-conducted systematic review (SR) on a topic in which there is scarce investment in primary studies. The publications found in the search justify carrying out an update of the SR on this topic. However, the authors should consider that the previous review has important flaws, since the search performed was not comprehensive (they included just the PUBMED database) and there was no assessment of the risk of bias. This suggests that the studies included in this previous review could be critically evaluated according to the protocol conducted by the authors. Or at least, these previous results should have been better explored since the objective was to update knowledge regarding the health of maritime workers.”

**Question**: Review the sentence “Poor oral health not only results in systemic diseases such as cardiovascular diseases, bacterial pneumonia, and diabetes mellitus, …” we know that there is an interrelationship between oral health and general health status, but this causal relationship is still under investigation and has been presented in a simplistic way.

**Answer**: thank you for your review, we have revised the introduction part based on your feedback (Line 55).

**Question**: Introduction - In the sentence “Nicotine from tobacco and fermentable carbohydrates can increase the risk of periodontal diseases and dental caries, respectively.” Add oral cancer and potentially malignant oral disorders related to smoking (leukoplakia and erythroplakia). And address other risk factors for oral lesions such as alcoholism and sun exposure (lower lip), since these conditions were the outcome of some studies included in the review.

**Answer**: We have included other associated risk factors to oral cancers and potentially malignant disorders in the introduction part. (Line 79)

**Question**: Introduction - In the sentence "To the best of our knowledge, there is a systematic review about the oral health of seafarer with underrepresented data about oral health in civilian sector and less developed countries." Perhaps because these primary studies did not exist at the time of this previous review...

**Answer**: thank you for the insights, it is true that many studies about oral health of fishermen and seafarer in developing countries have published after the period of 2010. We have revised this sentence based on your comment. (Line 95)

**Question**: Methods - The authors should adopt the updated version of PRISMA published in 2020.

**Answer**: we have updated to the latest version of Prisma flowchart and presented in S1 Figure.

**Question**: The databases cited in the methodology differ from those cited in the abstract.

**Answer**: we have changed to the database name to make it consistent with the methodology.

**Question**: Results - In the sentence "Of the 25 retrieved articles, six articles were excluded because of..." Articles excluded in phase 2 must be cited in the text or the list of the excluded references and justification must be provided in the appendices.

**Answer**: we thank the reviewer for this suggestion. We have updated the search and included in-text citations of all studies that were excluded in phase two. (Line 146)

**Question**: Results - In this sentence "In general, 13 selected scoring articles 6 to 9 were considered as having low risk of bias and acceptable quality. While five studies scored 2 to 5, which can be seen as having high risk of bias. When assessing the quality of the RCT, seven out of 11 items are fulfilled, and the quality of the RCT can be rated as moderate risk of bias.” The authors could cite the respective references as it is an important result of RS and needs to be easily accessible to the reader.

**Answer**: we have added respective references in this part. (Line 169)

**Question**: Results - In the sentence “The prevalence of smoking among seafarers was 25% and 56%, respectively.” Are these two prevalences from the studies mentioned later or is there any missing information?

**Answer**: thank you for your feedback, there was some missed data in this part, we have added more detail about prevalence of tobacco consumption (both smoking and smokeless) in this part. (Line 206)

**Question**: Discussion: It is unclear how the authors evaluated the data from the primary studies of high risk of bias in the analysis of evidence from the frequent oral conditions in the seafarers and fisherman.

**Answer**: we have evaluated the data from studies that have poor quality in this systematic review under the discussion part. (Line 298).
